# Association of the Pan-Immune-Inflammatory Value (PIV) and HALP score with disease prognosis and recovery in patients with acute pericarditis: An observational cohort study

**DOI:** 10.1097/MD.0000000000044460

**Published:** 2025-09-12

**Authors:** Hatice Eyiol, Azmi Eyiol, Ahmet Taha Sahin

**Affiliations:** a Department of Anesthesiology, Beyhekim Training and Research Hospital, Konya, Turkey; b Department of Cardiology, Beyhekim Training and Research Hospital, Konya, Turkey.

**Keywords:** HALP score, pericardial effusion, pericarditis, PIV, prognosis

## Abstract

Acute pericarditis is a sudden, typically noninfectious inflammation of the membrane surrounding the heart. It can arise from various causes, including viral infections, autoimmune diseases, cancer, trauma, or medications. This study aimed to evaluate the prognostic significance of the Pan-Immune-Inflammatory Value (PIV) and the hemoglobin, albumin, lymphocyte, and platelet (HALP) score in predicting disease severity, recurrence, and prognosis in patients with acute pericarditis. A retrospective cohort study was conducted, including 281 patients diagnosed with acute pericarditis between 2014 and 2023. Data on hemoglobin, albumin, neutrophil, monocyte, lymphocyte, and platelet levels, as well as clinical characteristics, treatment outcomes, and recurrence status, were extracted from hospital records. The PIV was calculated using the formula: neutrophil count (10³/mL) × monocyte percentage (%) × platelet count (10⁹/L)/lymphocyte count (10³/mL). The HALP score was determined as hemoglobin (g/L) × albumin (g/L) × lymphocyte count (10³/mL)/platelet count (10⁹/L). Statistical analyses were performed to evaluate the associations between these biomarkers and clinical outcomes. PIV was significantly higher in patients with pericardial effusion, pericardial friction rub, and persistent chest pain at 15-day follow-up (*P* = .003). Patients with hypertension, hyperlipidemia, and recent infections showed elevated PIV values, indicating more severe disease and a higher likelihood of recurrence. HALP score was significantly lower in patients with pericardial effusion and in nonsmokers. HALP score was significantly higher in patients with electrocardiography changes. PIV emerged as a stronger predictor of pericarditis severity, prognosis, and recurrence compared to the HALP score. The combined use of both markers may enhance the assessment of disease severity and prognosis, offering valuable insights for clinical decision-making in pericarditis management. Further studies are needed to validate these findings.

## 1. Introduction

Acute pericarditis is a sudden, typically noninfectious inflammation of the membrane surrounding the heart. It can arise from various causes, including viral infections, autoimmune diseases, cancer, trauma, or medications. Infections, particularly viral agents such as coxsackievirus, are the most common cause of pericarditis. In these cases, the virus can directly infect the pericardium or trigger inflammation through the immune response. Autoimmune diseases, where the body’s immune system attacks its own tissues, can also trigger pericarditis. Additionally, factors such as cancer or trauma may play a role in the development of pericarditis. Recurrence may occur due to untreated underlying causes, inadequate treatment, or the reemergence of triggers. However, in most cases, a specific cause cannot be identified.^[1–3]^

Criteria for hospital admission depend on the severity of pericarditis, the intensity of symptoms, and the identification of underlying causes. Typically, hospitalization is required for severe symptoms such as intense chest pain or shortness of breath, pericardial effusion, cardiac tamponade, or any situation in which pericarditis affects heart function. Additionally, individuals at higher risk for complications, particularly older adults or those with underlying health issues, may need hospital admission. Cases that fail to respond to treatment or develop serious complications should also be monitored in the hospital. However, some patients with mild symptoms and stable conditions may rest at home and undergo regular follow-up.^[1,4–6]^

The Pan-Immune-Inflammatory Value Index (PIV) is a biomarker that reflects the body’s overall inflammatory status.^[7,8]^ In patients with pericarditis, this marker may be crucial in assessing the severity and extent of the disease. Elevated PIV values are generally associated with more severe inflammation, leading to longer hospital stays. This study aims to evaluate the predictive utility of PIV in determining disease recurrence, severity, and prognosis based on a retrospective analysis of acute pericarditis patients treated at our institution.

Inflammation leads to a decrease in lymphocyte count while promoting the proliferation of platelets and neutrophils. Abnormal hemoglobin concentration is a known risk factor that increases the likelihood of adverse cardiovascular outcomes.^[9]^ Serum albumin levels are recognized as important biomarkers for various inflammatory processes and cardiovascular diseases.^[10]^ The hemoglobin, albumin, lymphocyte, and platelet (HALP) score, which incorporates these parameters, is increasingly used to assess overall nutritional status and systemic inflammation in patients.^[11]^ Evidence suggests that the HALP score has the ability to accurately predict mortality in individuals with coronary artery disease (CAD).^[12,13]^ Similarly, PIV, a novel inflammatory marker, has demonstrated its potential in predicting the severity of CAD.^[14]^ However, the prognostic utility of these 2 markers in patients with pericarditis remains unexplored in the literature. Therefore, this study aims to investigate the correlation between PIV, HALP score, and the prognosis and recovery process in patients with pericarditis.

## 2. Methods

### 2.1. Compliance with ethical standards

This study complies with all relevant national regulations, institutional policies, and the principles of the Declaration of Helsinki and has been approved by the Ethics Committee of Konya Necmettin Erbakan University Faculty of Medicine (approval number: 2024/4972). Written informed consent was obtained from all participants. Artificial intelligence-supported technologies were not used in the study.

### 2.2. Study design

This study was designed as a retrospective cohort study, evaluating a total of 281 patients. Data were obtained from hospital records for the analysis of patients diagnosed with acute pericarditis between 2014 and 2023. The data sources included the hospital’s electronic health records, laboratory results, electrocardiogram (ECG) reports, echocardiography findings, and treatment records. Patient records were retrospectively reviewed to collect information on hemoglobin, albumin, neutrophil, monocyte, lymphocyte, and platelet levels, as well as treatment methods, length of hospital stay, response to therapy, and recurrence status.

### 2.3. Patient evaluation and follow-up

Patients were evaluated based on demographic information (age, gender, presence of chronic diseases), clinical findings (chest pain, ECG findings, echocardiography results), and laboratory tests (hemoglobin, albumin, neutrophil, monocyte, lymphocyte, platelet, C-reactive protein, troponin levels). Treatment data, including medications used (NSAIDs, colchicine, corticosteroids), duration of treatment, treatment methods, and length of hospital stay, were collected. Outcome data such as recurrence cases, time to recurrence, and the need for additional treatment were also gathered. The glomerular filtration rate was calculated using the Cockcroft–Gault formula. The PIV index was determined using the formula: neutrophil count (10³/mL) × monocyte percentage (%) × platelet count (10⁹/L)/lymphocyte count (10³/mL). The HALP score was calculated as hemoglobin (g/L) × albumin (g/L) × lymphocyte count (10³/mL)/platelet count (10⁹/L).^[9,10]^

Demographic details, clinical findings, and diagnostic test results were meticulously recorded. Laboratory tests focused on hemoglobin, albumin, neutrophil, monocyte, lymphocyte, and platelet levels, along with other inflammatory markers. Blood samples were collected at the time of admission to the emergency department and cardiology outpatient clinics to assess white blood cell (WBC), neutrophil, monocyte, lymphocyte, platelet, hemoglobin, and albumin levels. Hematological parameters were measured using the Mindray BC-6800 automated hematology analyzer (Shenzhen, China), while albumin levels were analyzed using the Mindray BS-2000M chemistry analyzer (Shenzhen, China). Treatment data included the specific medications used, their duration, and the length of hospital stay. Outcome data highlighted recurrence rates, time to recurrence, and the need for additional treatment. Patients without a definitive diagnosis of acute pericarditis, those with missing key laboratory parameters (hemoglobin, albumin, neutrophil, monocyte, lymphocyte, and platelet), or those with other serious chronic conditions affecting these parameters (e.g., chronic kidney disease, rheumatic diseases, severe liver disease) were excluded. Additionally, patients with insufficient follow-up (less than 6 months), those under 18 years of age, those who were noncompliant with treatment, or those who did not complete therapy were excluded from the study.

### 2.4. Statistical analysis

Statistical analyses were performed using SPSS version 27.0 (IBM Inc., Chicago). The normality of distribution for numerical variables was assessed using the Kolmogorov–Smirnov test, histogram analysis, skewness/kurtosis data, and Q–Q plots. Descriptive statistics for both numerical and categorical variables were analyzed, with parameters expressed as IQR (median [minimum–maximum]) or mean ± SD. Relationships between 2 groups were examined using the Mann–Whitney *U* test or independent *t* test, as appropriate. Correlations between quantitative variables were evaluated using Spearman correlation analyses. Throughout the study, a type I error rate of 5% (α = 0.05) was considered, and a *P*-value of < .05 was regarded as the threshold for statistical significance.

## 3. Results

In our study, the overall distribution of quantitative parameters in pericarditis patients is summarized in Table 1. The median age was 41 years, ranging from 18 to 65. The median WBC count was 9.74 × 10³/mL, median lymphocyte count was 2.26 × 10³/mL, and median neutrophil count was 6.45 × 10³/mL, while the mean platelet count was 255.0 ± 55.0 × 10³/mL. The mean hemoglobin level was 14.3 ± 1.5 (8.9–17.7) g/dL and the mean albumin level was 42.8 ± 3.4 (32–51) g/L. Other parameters, such as C-reactive protein and troponin levels, exhibited a wide range of values, reflecting the patients’ diverse inflammatory and cardiac profiles.

**Table 1:** Summary of the general distribution of quantitative parameters in pericarditis patients.

Parameters	Unit	Minimum	Maximum	Distribution*
Age	yr	18.00	65.00	41 (18–65)
WBC	10^3^/mL	8.01	23.82	9.74 (8.01–23.82)
Neutrophil	10^3^/mL	0.33	19.93	6.45 (0.33–19.93)
Monocyte	%	0.04	1.50	0.54 (0.04–1.5)
Lenphocyte	10^3^/mL	0.51	4.78	2.26 (0.51–4.78)
Hemoglobin	g/dL	8.90	17.70	14.3 ± 1.5
Platelet	10^3^/mL	135.0	464.0	255.0 ± 55.0
RDW	%	11.20	17.70	13.5 ± 1.0
Albumin	g/L	32.00	51.00	42.8 ± 3.4
D-dimer	ng/mL	117.0	809.0	482.0 ± 101.0
LDL	mg/dL	33.0	200.0	131.0 ± 28.0
HDL	mg/dL	27.0	65.0	44.0 ± 6.0
Triglyceride	ng/dL	61.0	606.0	128 (61–606)
ASO	IU/mL	34.0	345.0	154 (34–345)
Troponin	ng/L	3.0	36.0	11 (3–36)
CRP	mg/L	4.0	203.0	23 (4–203)
Ferritin	ng/mL	19.0	262.0	67 (19–262)
Fibrinogen	ng/dL	2.43	5.58	3.1 (2.43–5.58)
Uric acid	mg/dL	3.70	6.10	4.9 (3.7–6.1)
Glucose	mg/dL	78.0	287.0	95 (78–287)
PIV		7.96	4146.0	408.7 (7.96–4146.0)
HALP score		1.30	18.88	5.46 (1.30–18.88)
EF	%	30.0	65.0	60 (30–65)

ASO = antistreptolysin O, CRP = C-reactive protein, EF = ejection fraction, HDL = high density lipoprotein, LDL = low density lipoprotein, PIV = Pan-Immune-Inflammatory-Value Index, RDW = red cell distribution width, TRG = triglyceride, TyG Index = Triglyceride–Glucose Index, WBC = white blood cell.

* Parameters are expressed as IQR (interquartile range) [median, min, and max] or mean±SD.

The comparison of parameters by gender is shown in Table 2. Significant differences were observed between male and female patients in terms of platelet count, hemoglobin level, albumin level, platelet level, HDL level, HALP score level, and ejection fraction (EF). Males had higher hemoglobin, albumin and HALP score levels, while females had higher platelet counts, HDL levels, and EF (Table 2). These differences suggest potential gender-based variations in the inflammatory response and disease severity in acute pericarditis.

**Table 2:** Comparison of parameters according to gender in pericarditis patients.

		Sex		*P*
		Male (n = 158, 56.2%)	Female (n = 123, 43.8%)	
Parameters	Unit	Distribution*		
Age	yr	41 (19–65)	41 (18–65)	.120†
WBC	10^3^/mL	9.73 (8.01–19.98)	9.78 (8.05–23.82)	.644†
Neutrophil	10^3^/mL	6.28 (3.62–16.29)	6.64 (0.33–19.93)	.186†
Monocyte	%	0.56 (0.04–1.5)	0.54 (0.18–1.37)	.748†
Lenphocyte	10^3^/mL	2.24 (0.58–4.78)	2.3 (0.51–4.02)	.969†
Hemoglobin	g/dL	15.1 ± 1.3	13.4 ± 1.3	**<.001** ‡
Platelet	10^3^/mL	245.0 ± 49.0	267.0 ± 61.0	**.002** ‡
RDW	%	13.5 ± 0.9	13.4 ± 1.2	.379‡
Albumin	g/L	43.8 ± 2.9	41.6 ± 3.6	**<.001** ‡
D-dimer	ng/mL	486 ± 88	476 ± 116	.402‡
LDL	mg/dL	132.0 ± 28.0	131.0 ± 27.0	.710‡
HDL	mg/dL	43.0 ± 6.0	46.0 ± 5.0	**<.001** ‡
Triglyceride	ng/dL	127 (61–606)	133 (67–348)	.849†
ASO	IU/mL	155 (50–305)	145 (34–345)	.283†
Troponin	ng/L	11 (3–36)	10 (3–29)	.138†
CRP	mg/L	23 (4–203)	24 (4–203)	.219†
Ferritin	ng/mL	67 (19–262)	71 (24–144)	.314†
Fibrinogen	ng/dL	3.11 (2.43–5.58)	3.09 (2.5–4.07)	.857†
Uric acid	mg/dL	4.8 (3.7–6.1)	5.1 (3.7–6.1)	.057†
Glucose	mg/dL	95 (79–280)	96 (78–287)	.222†
PIV		405.12 (7.96–2760.25)	411.49 (41.25–4146.6)	.116†
HALP score		6.28 (1.3–18.88)	4.63 (1.37–11.47)	**<.001** †
EF	%	60 (30–65)	60 (50–65)	**<.001** †

Bold values show statistically significant values.

ASO = antistreptolysin O, CRP = C-reactive protein, EF = ejection fraction, HDL = high density lipoprotein, LDL = low density lipoprotein, PIV = Pan-Immune-Inflammatory-Value Index, RDW = red cell distribution width, TRG = triglyceride, TyG Index = Triglyceride–Glucose Index, WBC = white blood cell.

* Parameters are expressed as IQR (interquartile range) [median, min, and max] or mean±SD.

† Mann–Whitney *U* test.

‡ Independent *t* test.

Table 3 presents the HALP score according to specific clinical assessment. HALP score was significantly lower in patients with pericardial effusion and in nonsmokers. HALP score was significantly higher in patients with ECG changes. There was no significant difference in HALP score between those with ongoing pain and those with resolution of pain at 15-day follow-up (*P* = .051). These findings suggest that a lower HALP score is associated with more severe disease manifestations such as pericardial effusion.

**Table 3:** Comparison of HALP score values in pericarditis patients according to the presence of specific conditions.

		HALP Score	*P* *
Parameters		Median (min–max)	
Pericardial effusion	No (n = 147)	6.06 (1.54–18.88)	**<.001**
	Yes (n = 134)	4.39 (1.3–14.12)	
ECG change	No (n = 77)	4.36 (1.37–9.59)	**<.001**
	Yes (n = 204)	5.83 (1.3–18.88)	
Pericardial frontman	No (n = 266)	5.51 (1.3–18.88)	.392
	Yes (n = 15)	4.63 (3.58–8.89)	
IV steroid	No (n = 263)	5.53 (1.3–18.88)	.051
	Yes (n = 18)	3.98 (2.71–9.31)	
15th day control	Healing (n = 263)	5.53 (1.3–18.88)	.051
	Continue Pain (n = 18)	3.98 (2.71–9.31)	
Hypertension	No (n = 161)	5.46 (1.3–14.12)	.825
	Yes (n = 120)	5.46 (1.37–18.88)	
Hyperlipidemia	No (n = 217)	5.53 (1.3–14.12)	.268
	Yes (n = 64)	5.04 (1.37–18.88)	
Diabetes mellitus	No (n = 246)	5.51 (1.3–16.32)	.399
	Yes (n = 35)	5.18 (2.66–18.88)	
Smoking	No (n = 122)	4.78 (1.37–13.87)	**<.001**
	Yes (n = 159)	6.12 (1.3–18.88)	
Family history	No (n = 220)	5.39 (1.3–16.32)	.849
	Yes (n = 61)	5.78 (2.71–18.88)	
Obesity	No (n = 122)	5.78 (1.3–14.12)	.125
	Yes (n = 159)	5.17 (1.37–18.88)	
Flu infection within 4 wk	No (n = 63)	5.46 (2.9–9.62)	.461
	Yes (n = 218)	5.46 (1.3–18.88)	
Tonsillitis	No (n = 208)	5.53 (1.3–18.88)	.363
	Yes (n = 73)	5.32 (2.9–9.46)	
Gastroenteritis within 4 wk	No (n = 234)	5.68 (1.3–18.88)	.417
	Yes (n = 47)	4.93 (1.37–16.32)	

Bold values show statistically significant values.

ECG = electrocardiography, IV = intravenous.

* Mann–Whitney *U* test.

Table 4 evaluates the PIV value according to specific clinical conditions. The PIV was significantly higher in patients with pericardial effusion, those with a pericardial friction rub, and those receiving intravenous steroid therapy. Additionally, patients with persistent pain at the 15-day follow-up had significantly higher PIV values compared to those who had recovered (*P* = .003). The PIV was also significantly elevated in patients with a diagnosis of hypertension, diabetes mellitus, hyperlipidemia or obesity, a history of tonsillitis before the diagnosis of pericarditis, and those who had gastroenteritis within 3 to 4 weeks before diagnosis. These findings suggest that a higher PIV may be associated with more severe disease and a greater likelihood of recurrence.

**Table 4:** Comparison of PIV values in pericarditis patients according to the presence of specific conditions.

		PIV	*P* *
Parameters		Median (min–max)	
Pericardial effusion	No (n = 147)	396.09 (7.96–1776.01)	**.045**
	Yes (n = 134)	426.63 (41.25–4146.6)	
ECG change	No (n = 77)	382.6 (41.25–2760.25)	.608
	Yes (n = 204)	410.41 (7.96–4146.6)	
Pericardial frontman	No (n = 266)	403.86 (7.96–4146.6)	**.003**
	Yes (n = 15)	678.44 (217.59–1791.76)	
IV steroid	No (n = 263)	403.31 (7.96–4146.6)	**.003**
	Yes (n = 18)	678.44 (187.76–1791.76)	
15th day control	Healing (n = 263)	403.31 (7.96–4146.6)	**.003**
	Continue Pain (n = 18)	678.44 (187.76–1791.76)	
Hypertension	No (n = 161)	432.86 (140.86–4146.6)	**<.001**
	Yes (n = 120)	359.59 (7.96–2489.91)	
Hyperlipidemia	No (n = 217)	422.12 (106.53–4146.6)	**.004**
	Yes (n = 64)	337.43 (7.96–1472.9)	
Diabetes mellitus	No (n = 246)	412.53 (106.53–4146.6)	**.036**
	Yes (n = 35)	358.35 (7.96–1472.9)	
Smoking	No (n = 122)	408.97 (41.25–4146.6)	.341
	Yes (n = 159)	408.28 (7.96–2760.25)	
Family history	No (n = 220)	408.97 (41.25–4146.6)	.599
	Yes (n = 61)	401.79 (7.96–2489.91)	
Obesity	No (n = 122)	434.47 (140.86–2760.25)	**.005**
	Yes (n = 159)	373.82 (7.96–4146.6)	
Flu infection within 4 wk	No (n = 63)	439.56 (187.76–1791.76)	.061
	Yes (n = 218)	395.39 (7.96–4146.6)	
Tonsillitis	No (n = 208)	380.86 (7.96–4146.6)	**<.001**
	Yes (n = 73)	542.31 (187.76–1923.29)	
Gastroenteritis within 4 wk	No (n = 234)	426.63 (7.96–4146.6)	**.002**
	Yes (n = 47)	343.42 (126.23–1072.8)	

Bold values show statistically significant values.

ECG = electrocardiography, IV = intravenous.

* Mann–Whitney *U* test.

**Figure 1.**
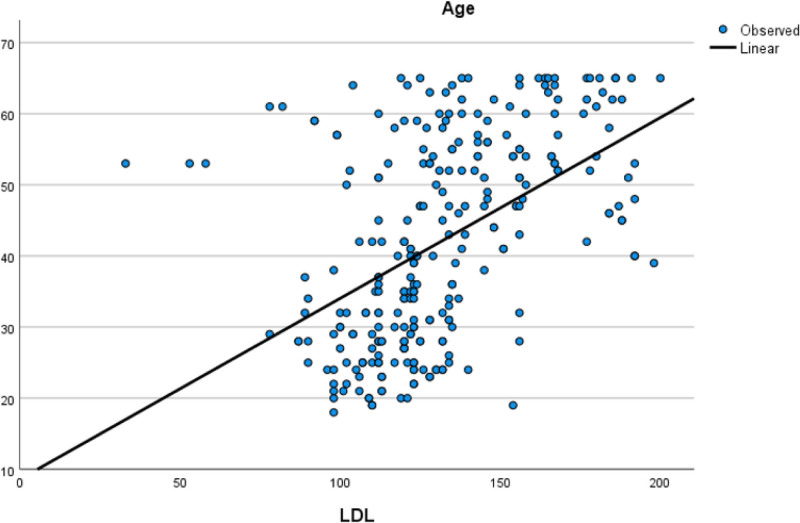
Moderate positive correlation relationship between age and LDL. LDL = low density lipoprotein.

**Figure 2.**
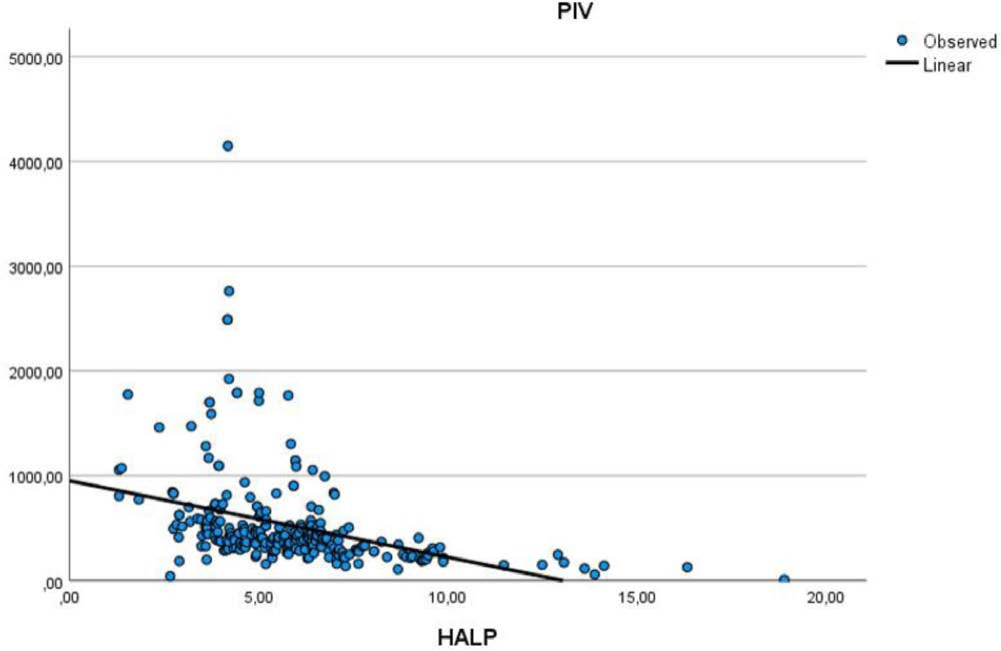
Moderate negative correlation relationship between PIV and HALP score. HALP score = hemoglobin, albumin, lymphocyte, and platelet, PIV = Pan-Immun-Inflammatory-Value Index.

**Figure 3.**
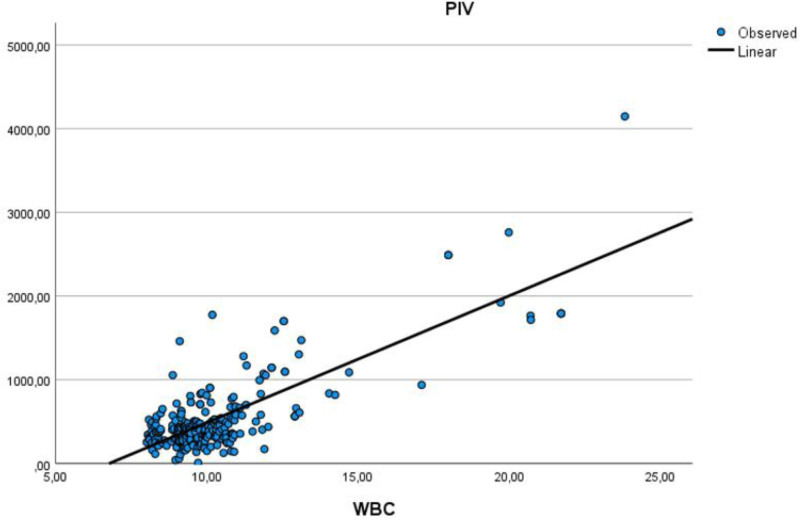
Moderate positive correlation relationship between PIV and WBC. PIV = Pan-Immun-Inflammatory-Value Index, WBC = white blood cell.

**Figure 4.**
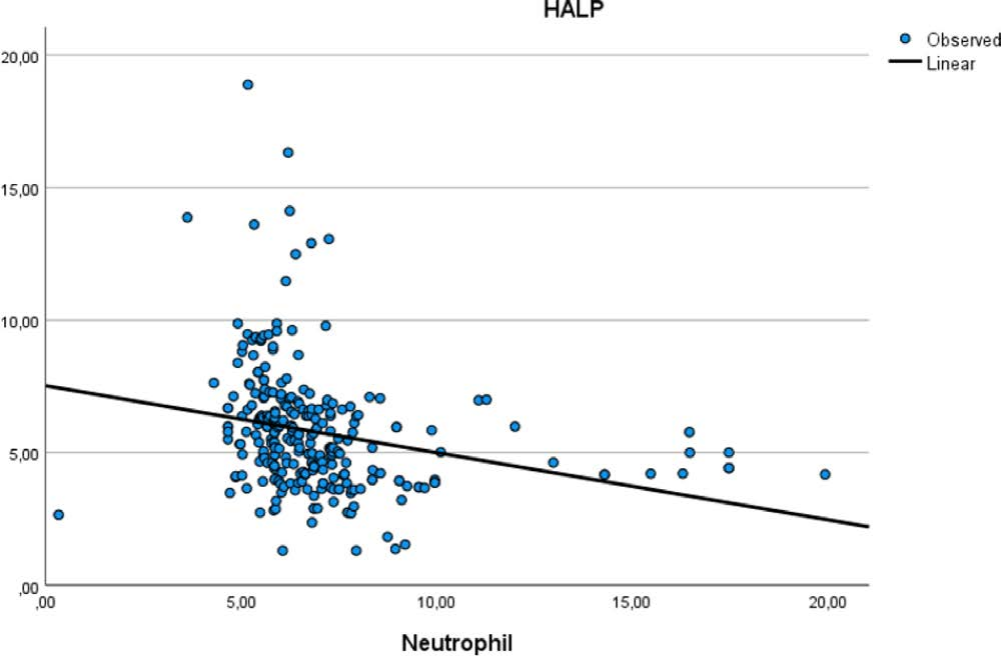
Moderate negative correlation relationship between HALP score and neutrophil levels. HALP score = hemoglobin, albumin, lymphocyte, and platelet.

Table 5 assesses the correlations between age, HALP score, PIV, EF, and other parameters. There was a moderate positive correlation between age and low density lipoprotein, while a moderate negative correlation was observed between age and HDL. Additionally, a moderate negative correlation was found between HALP score and PIV, and HALP score and WBC. A moderate positive correlation was noted between PIV and WBC count. The moderate positive correlation relationship between age and low density lipoprotein is illustrated in Figure 1. The moderate negative correlation relationship between PIV and HALP score is illustrated in Figure 2. The moderate positive correlation relationship between PIV and WBC is illustrated in Figure 3. The moderate negative correlation relationship between HALP score and neutrophil levels is illustrated in Figure 4.

**Table 5:** To examine the correlation relationships of age, TyG Index, PIV, and EF with other parameters and with each other.

		Age	PIV	HALP score	EF (%)
Age	rho	–	-0.197	-0.099	-0.353
	*P*	–	**.001**	.097	**<.001**
PIV	rho	-0.197	–	-0.555	0.195
	*P*	**.001**	–	**<.001**	**.001**
HALP score	rho	-0.099	-0.555	–	-0.073
	*P*	.097	**<.001**	–	.224
EF	rho	-0.353	0.195	-0.073	–
	*P*	**<.001**	**.001**	.224	–
WBC	rho	-0.148	0.453	-0.098	0.113
	*P*	**.013**	**<.001**	.1	.059
Neutrophil	rho	-0.126	–	-0.415	0.093
	*P*	**.035**	–	**<.001**	.119
Monocyte	rho	-0.242	–	0.106	0.185
	*P*	**<.001**	–	.077	**.002**
Lymphocyte	rho	-0.106	–	–	.047
	*P*	.075	–	–	.431
Hemoglobin	rho	-0.058	-0.164	–	-.119
	*P*	.331	**.006**	–	**.046**
Platelet	rho	-0.045	–	–	.1
	*P*	.453	–	–	.094
RDW	rho	0.154	0.041	-0.122	-.137
	*P*	**.01**	.497	**.041**	**.022**
Albumin	rho	-0.055	-0.165	–	-.086
	*P*	.354	**.006**	–	.15
D-dimer	rho	0.064	0.11	-0.02	-.117
	*P*	.283	.065	.732	.05
LDL	rho	0.541	-0.171	0.025	-.287
	*P*	**<.001**	**.004**	.673	**<.001**
HDL	rho	-0.429	0.206	-0.069	.178
	*P*	**<.001**	**.001**	.248	**.003**
Triglyceride	rho	0.364	-0.138	0.005	-.279
	*P*	**<.001**	**.02**	.93	**<.001**
ASO	rho	-0.27	0.068	0.074	-.074
	*P*	**<.001**	.254	.216	.216
Troponin	rho	0.076	0.05	-0.144	-.061
	*P*	.202	.407	**.015**	.308
CRP	rho	-0.353	0.347	-0.257	.202
	*P*	**<.001**	**<.001**	**<.001**	**.001**
Ferritin	rho	-0.313	0.228	-0.177	.127
	*P*	**<.001**	**<.001**	**.003**	**.033**
Fibrinogen	rho	0.181	0.072	-0.087	-.172
	*P*	**.002**	.226	.147	**.004**
Uric acid	rho	0.082	0.097	-0.255	.01
	*P*	.17	.104	**<.001**	.864
Glucose	rho	0.389	<0.001	-0.165	-.142
	*P*	**<.001**	.996	**.006**	**.017**

Bold values show statistically significant values.

Spearman correlation analysis (correlation coefficient = rho).

ASO = antistreptolysin O, CRP = C-reactive protein, EF = ejection fraction, HDL = high density lipoprotein, LDL = low density lipoprotein, PIV = Pan-Immun-Inflammatory-Value Index, RDW = red cell distribution width, TRG = triglyceride, TyG Index = Triglyceride–Glucose Index, WBC = white blood cell.

## 4. Discussion

Acute pericarditis is a sudden, typically noninfectious inflammation of the pericardium. It can result from various causes, including viral infections, autoimmune diseases, malignancies, trauma, or medications. Infections, particularly viral agents such as coxsackievirus, are the most common cause of pericarditis. In these cases, the virus may directly infect the pericardium or trigger inflammation through an immune response. Autoimmune diseases, in which the body’s immune system attacks its own tissues, can also contribute to the development of pericarditis.^[1–3]^

Inflammation and coagulation are closely interconnected and mutually dependent processes. Neutrophils, in particular, play a crucial role in the development of atherosclerosis and thrombus formation. While neutrophil counts reflect the duration of inflammation, lymphocyte counts represent specific mechanisms involved in immune regulation.^[14]^ Inflammatory processes lead to an increase in neutrophil, monocyte, and platelet counts while simultaneously reducing lymphocyte levels.^[15]^ A decline in lymphocytes following acute inflammation has been associated with worse cardiovascular outcomes. Additionally, platelets contribute to both the acute and chronic inflammatory processes of CAD.^[12]^

The relationship between the PIV and disease severity, prognosis, and recurrence rates in pericarditis patients is a multifaceted topic that warrants further investigation. In our study, PIV was found to be significantly higher in patients with pericardial effusion, those with pericardial friction rub, and those treated with intravenous steroids. These findings suggest that PIV may be influenced by both the clinical presentation of pericarditis and the therapeutic interventions used. Notably, PIV was observed to be elevated in patients who continued to experience chest pain 15 days after treatment, indicating that patients with higher PIV values may have a prolonged recovery period and an increased likelihood of recurrence. Elevated PIV was also commonly observed in patients with a history of tonsillitis related to an infectious etiology and in those who had gastroenteritis 3 to 4 weeks prior to the pericarditis diagnosis. Additionally, the presence of hypertension and hyperlipidemia was associated with increased PIV, suggesting that these comorbidities may contribute to an exacerbated inflammatory response.

Previous studies have emphasized the prognostic value of inflammatory markers in pericarditis. For example, Wu et al demonstrated that elevated PIV was associated with worse outcomes in various cardiovascular conditions, reflecting an underlying heightened inflammatory state.^[16]^ Our findings align with this paradigm, suggesting that higher PIV values may reflect a more severe inflammatory response in pericarditis, potentially leading to increased disease severity, prolonged recovery, and a higher likelihood of recurrence.

There was a moderate correlation between PIV and WBC count. This relationship highlights the potential role of PIV as a surrogate marker of systemic inflammation and immune response. The PIV, an index incorporating 4 key hematological parameters (neutrophil, monocyte, platelet, and lymphocyte counts) has been recently developed as a marker for assessing the severity of inflammation.^[15,17–25]^ Recent studies have confirmed the predictive significance of PIV in various inflammatory diseases.^[26–28]^ A study conducted on patients with ST-segment elevation myocardial infarction highlighted that PIV has superior mortality predictive ability compared to the neutrophil-to-lymphocyte ratio and platelet-to-lymphocyte ratio.^[7]^ Additionally, a study by Wu et al demonstrated that PIV is a reliable marker for predicting cardiovascular mortality.^[16]^ Çetinkaya et al reported that despite a PIV cutoff value of 568.2, it could predict severe coronary lesions with 91% sensitivity and 81.1% specificity.^[14]^ The findings of Özcan Çetin et al^[29]^ and Kalyoncuoğlu et al^[30]^ , which demonstrated that elevated WBC and PIV levels were indicative of severe inflammatory processes, further support our results.^[31,32]^

Numerous studies in the literature have concluded that the novel HALP score may be a valuable biomarker for the diagnosis and prognosis of various inflammation-related conditions.^[17–20]^ As previously established, hemoglobin and albumin levels reflect the body’s nutritional status, while lymphocytes and platelets are associated with immune function.^[11]^ Inflammation reduces albumin synthesis, while increased cytokine levels inhibit erythrocyte maturation, potentially leading to anemia.^[12]^ Prognosis in cardiovascular disease has been linked to malnutrition and anemia.^[21]^ Albumin plays a crucial role in cardiovascular health due to its anti-inflammatory, antioxidant, and antithrombotic properties.^[33]^ Research has demonstrated a correlation between lower albumin levels and an increased risk of atrial fibrillation (AF).^[22]^ Bonde et al reported that 13% to 34% of patients with non-valvular AF were anemic, which may indicate a poor prognosis.^[9]^

Recent studies suggest that the HALP score may also be used to predict mortality in individuals with cardiovascular disease.^[23,24]^ According to Zheng et al, patients with a HALP score ≤ 69.68 had a higher risk of all-cause mortality. Furthermore, the HALP score had a sensitivity of 0.510, a specificity of 0.654, and an AUC of 0.610 for predicting cardiovascular disease prognosis, outperforming albumin, lymphocyte, and platelet levels alone.^[12]^ Karakayali et al conducted a study on patients with CAD and found that the HALP score was independently associated with in-hospital mortality, as determined by Cox proportional hazards analysis.^[13]^ In our other study investigating the prognostic significance of PIV and HALP scores in critically ill patients with and without AF, both scores were significantly associated with AF. While the HALP score proved to be a strong prognostic marker in these patients, the predictive ability of PIV for mortality was found to be insufficient.^[34]^

In our study, the HALP score was significantly lower in patients with pericardial effusion. These findings suggest that the HALP score may be influenced by both the clinical presentation of pericarditis and the therapeutic interventions employed. The lack of a significant difference in HALP score among patients with pericardial friction rub, those receiving IV steroids, and those with persistent chest pain 15 days after treatment suggests that additional inflammatory markers may be needed to assess these clinical situations.

Methodologically, PIV and HALP score can be easily calculated based on routine blood biochemistry tests in clinical practice worldwide. These accessible and straightforward methods could provide valuable insights into the prognosis, clinical status, and recurrence rates in patients with pericarditis. However, further studies are needed to confirm the clinical utility of these parameters. Additional research should also consider whether factors such as the ethnicity of participants, comorbid conditions, and follow-up duration influence outcomes.

### 4.1. Limitations

This study has several limitations that should be considered. First, its retrospective design may introduce biases related to data collection and patient selection, potentially affecting the generalizability of the findings. Second, although the sample size was adequate, it may not fully capture the variability present in the broader population of pericarditis patients, limiting the external validity of the results. Additionally, the study was based on data from a single center, which may not reflect differences in clinical practices or patient characteristics in other settings. Also, the single-center nature of our study limits the generalizability of our results; future multicenter studies are required to validate these findings in broader and more diverse populations. Finally, while PIV and HALP score show promise as prognostic markers, the lack of prospective validation and integration with other clinical variables highlights the need for further research to confirm these findings and assess their practical utility in routine clinical practice.

## 5. Conclusion

In our study, PIV was shown to be a more significant predictor of pericarditis severity, prognosis, and recurrence rates compared to HALP score. The combined assessment of these 2 parameters could provide more valuable insights into patient prognosis and disease severity. Our findings highlight the potential of these markers to complement existing diagnostic and prognostic tools, contributing to a more comprehensive understanding of the disease.

## Author contributions

**Conceptualization:** Azmi Eyiol.

**Data curation:** Hatice Eyiol.

**Funding acquisition:** Ahmet Taha Sahin.

**Investigation:** Ahmet Taha Sahin.

**Methodology:** Hatice Eyiol.

**Resources:** Ahmet Taha Sahin.

**Software:** Ahmet Taha Sahin.

**Supervision:** Azmi Eyiol.

**Validation:** Ahmet Taha Sahin.

**Visualization:** Ahmet Taha Sahin.

**Writing – original draft:** Hatice Eyiol.

**Writing – review & editing:** Azmi Eyiol.

## References

[1] Spotts PH Zhou F. Myocarditis and pericarditis. Prim Care. 2024;51:111–24. 38278565 10.1016/j.pop.2023.07.006

[2] Boulet J Sridhar VS Bouabdallaoui N Tardif J-C White M. Inflammation in heart failure: pathophysiology and therapeutic strategies. Inflamm Res. 2024;73:709–23. 38546848 10.1007/s00011-023-01845-6 PMC11058911

[3] Adler Y Charron P Imazio M. 2015 ESC guidelines for the diagnosis and management of pericardial diseases: the task force for the diagnosis and management of pericardial diseases of the European society of cardiology. Eur Heart J. 2015;36:2921–64.26320112

[4] Lazaros G Antonopoulos AS Lazarou E. Age- and sexbased differences in patients with acute pericarditis. Eur J Clin Invest. 2021;51:e13392. 32857868 10.1111/eci.13392

[5] Lazarou E Lazaros G Antonopoulos AS. A risk score for pericarditis recurrence. Eur J Clin Invest. 2021;51:e13602. 34050527 10.1111/eci.13602

[6] Imazio M Spodick DH Brucato A Trinchero R Adler Y. Controversial issues in the management of pericardial diseases. Circulation. 2010;121:916–28. 20177006 10.1161/CIRCULATIONAHA.108.844753

[7] Murat B Murat S Ozgeyik M Bilgin M. Comparison of pan‐immune‐inflammation value with other inflammation markers of long‐term survival after ST‐segment elevation myocardial infarction. Eur J Clin Invest. 2023;53:e13872. 36097823 10.1111/eci.13872

[8] Kazan DE Kazan S. Systemic immune inflammation index and pan-immune inflammation value as prognostic markers in patients with idiopathic low and moderate risk membranous nephropathy. Eur Rev Med Pharmacol Sci. 2023;27:642–8 36734708 10.26355/eurrev_202301_31065

[9] Bonde AN Blanche P Staerk L. Oral anticoagulation among atrial fibrillation patients with anaemia: an observational cohort study. Eur Heart J. 2019;40:3782–90. 30932145 10.1093/eurheartj/ehz155

[10] Yuan HJ Zhong X Li Y Xue YT Jiao HC. Correlation between serum albumin and D-dimer levels in 909 patients with non-valvular atrial fibrillation: a retrospective study from a single center in China. Med Sci Monit. 2022;28:e938511. 36424830 10.12659/MSM.938511 PMC9707042

[11] Kocaoglu S Alatli T. The efficiency of the HALP score and the modified HALP score in predicting mortality in patients with acute heart failure presenting to the emergency department. J Coll Physicians Surg Pak. 2022;32:706–11. 35686400 10.29271/jcpsp.2022.06.706

[12] Zheng Y Huang Y Li H. Hemoglobin albumin lymphocyte and platelet score and all-cause mortality in coronary heart disease: a retrospective cohort study of NHANES database. Front Cardiovasc Med. 2023;10:1241217. 38028472 10.3389/fcvm.2023.1241217 PMC10679332

[13] Karakayali M Omar T Artac I. The prognostic value of HALP score in predicting in-hospital mortality in patients with ST-elevation myocardial infarction undergoing primary percutaneous coronary intervention. Coron Artery Dis. 2023;34:483–8. 37799045 10.1097/MCA.0000000000001271

[14] Cetinkaya Z Kelesoglu S Tuncay A. The role of pan-immuneinflammation value in determining the severity of coronary artery disease in NSTEMI patients. J Clin Med. 2024;13:1295. 38592192 10.3390/jcm13051295 PMC10931938

[15] Şen Uzeli U Başaran PO. Pan-immune inflammation value as a biomarker in ankylosing spondilitis and associated with disease activity. Anatolian Curr Med J. 2024;6:48–54.

[16] Wu B Zhang C Lin S Zhang Y Ding S Song W. The relationship between the pan-immune-inflammation value and long-term prognoses in patients with hypertension: National Health and Nutrition Examination Study, 1999–2018. Front Cardiovasc Med. 2023;10:1099427. 36937901 10.3389/fcvm.2023.1099427 PMC10017977

[17] Sargin ZG Dusunceli I. The effect of HALP score on the prognosis of gastric adenocarcinoma. J Coll Physicians Surg Pak. 2022;32:1154–9. 36089712 10.29271/jcpsp.2022.09.1154

[18] Ustaoglu M Aktas G Kucukdemirci O Goren I Bas B. Could a reduced hemoglobin, albumin, lymphocyte, and platelet (HALP) score predict autoimmune hepatitis and degree of liver fibrosis. Rev Assoc Med Bras (1992). 2024;70:e20230905. 38294124 10.1590/1806-9282.20230905 PMC10830098

[19] Bildik B Çekmen B Atiş SE Günaydin YK Dorter M. Evaluation of the relationship between Hemoglobin, Albumin, Lymphocyte, Platelet (HALP) score and treatment modality and mortality in patients with ileus. Ulus Travma Acil Cerrahi Derg. 2023;29:1351–6. 38073459 10.14744/tjtes.2023.68620 PMC10767295

[20] Zuo L Dong Y Liao X. Low HALP (Hemoglobin, Albumin, Lymphocyte, and Platelet) score increases the risk of post-stroke cognitive impairment: a multicenter cohort stud. Clin Interv Aging. 2024;19:81–92. 38223135 10.2147/CIA.S432885 PMC10788070

[21] Xiang W Chen X Ye W Li J Zhang X Xie D. Prognostic nutritional index for predicting 3-month outcomes in ischemic stroke patients undergoing thrombolysis. Front Neurol. 2020;11:599. 32670192 10.3389/fneur.2020.00599 PMC7333017

[22] Wang Y Du P Xiao Q. Relationship between serum albumin and risk of atrial fibrillation: a dose-response meta-analysis. Front Nutr. 2021;8:728353. 34490334 10.3389/fnut.2021.728353 PMC8418186

[23] Liu L Gong B Wang W Xu K Wang K Song G. Association between hemoglobin, albumin, lymphocytes, and platelets and mortality in patients with heart failure. ESC Heart Fail. 2024;11:1051–60. 38243382 10.1002/ehf2.14662 PMC10966267

[24] Pan H Lin S. Association of hemoglobin, albumin, lymphocyte, and platelet score with risk of cerebrovascular, cardiovascular, and all-cause mortality in the general population: results from the NHANES 1999–2018. Front Endocrinol (Lausanne). 2023;14:1173399. 37424853 10.3389/fendo.2023.1173399 PMC10328756

[25] Turan YB. The prognostic importance of the pan-immune-inflammation value in patients with septic shock. BMC Infect Dis. 2024;24:69. 38200436 10.1186/s12879-023-08963-w PMC10777599

[26] Hai-Jing Y Shan R Jie-Qiong X. Prognostic significance of the pretreatment pan-immune-inflammation value in cancer patients: an updated meta-analysis of 30 studies. Front Nutr. 2023;10:1259929. 37850085 10.3389/fnut.2023.1259929 PMC10577316

[27] Ocak T Lermi N Yilmaz BZ. Pan-immune-inflammation value could be a new marker to differentiate between vascular Behçet’s disease and non-vascular Behçet’s disease. Eur Rev Med Pharmacol Sci. 2024;28:1751–9. 38497857 10.26355/eurrev_202403_35588

[28] Zhang F Li L Wu X. Pan-immune-inflammation value is associated with poor prognosis in patients undergoing peritoneal dialysis. Ren Fail. 2023;45:2158103. 36632816 10.1080/0886022X.2022.2158103 PMC9848369

[29] Cetin EH Cetin MS Canpolat U. Monocyte/HDL-cholesterol ratio predicts the definite stent thrombosis after primary percutaneous coronary intervention for ST-segment elevation myocardial infarction. Biomark Med. 2015;9:967–77. 26439248 10.2217/bmm.15.74

[30] Oflar E Kalyoncuoğlu M Koyuncu A. The role of the inflammatory prognostic index in patients with non-ST elevation myocardial infarction undergoing percutaneous coronary intervention. J Clin Med. 2025;14:4491. 40648869 10.3390/jcm14134491 PMC12249563

[31] Ozcan Cetin EH Cetin MS Aras D. Platelet to lymphocyte ratio as a prognostic marker of in-hospital and long-term major adverse cardiovascular events in ST-segment elevation myocardial infarction. Angiology. 2016;67:336–45. 26101368 10.1177/0003319715591751

[32] Kalyoncuoglu M Durmus G. Relationship between C-reactive protein-to-albumin ratio and the extent of coronary artery disease in patients with non-ST-elevated myocardial infarction. Coron Artery Dis. 2020;31:130–6. 31233399 10.1097/MCA.0000000000000768

[33] Pan LY Song J. Association of red cell distribution width/albumin ratio and in hospital mortality in patients with atrial fibrillation base on medical information mart for intensive care IV database. BMC Cardiovasc Disord. 2024;24:174. 38515030 10.1186/s12872-024-03839-6 PMC10956318

[34] Eyiol A. The relationship of pan-immune-inflammation value (PIV) and HALP score with prognosis in patients with atrial fibrillation. Medicine (Baltimore). 2024;103:e39643. 39252218 10.1097/MD.0000000000039643 PMC11383257

